# 5-Fluorouracil Inhibits Bacterial Growth and Reduces Biofilm in Addition to Having Synergetic Effects with Gentamicin Against *Pseudomonas aeruginosa*

**DOI:** 10.3390/microorganisms12112257

**Published:** 2024-11-07

**Authors:** Amani A. Niazy, May M. Alrashed, Rhodanne Nicole A. Lambarte, Abdurahman A. Niazy

**Affiliations:** 1 Department of Clinical Laboratory Sciences, College of Applied Medical Sciences, King Saud University, Riyadh 12372, Saudi Arabia; aniazy@ksu.edu.sa (A.A.N.);; 2 Molecular and Cell Biology Laboratory, Prince Naif bin Abdulaziz Health Research Center, College of Dentistry, King Saud University Medical City, King Saud University, Riyadh 11545, Saudi Arabia; rlambarte@ksu.edu.sa; 3 Department of Oral Medicine and Diagnostic Sciences, College of Dentistry, King Saud University, Riyadh 11545, Saudi Arabia

**Keywords:** 5-fluorouracil, *Pseudomonas aeruginosa*, biofilm, growth inhibition, synergism

## Abstract

*Pseudomonas aeruginosa* is a multidrug-resistant pathogen known for chronic infections, mainly due to biofilm formation. This study aimed to explore the potential repurposing of 5-fluorouracil (5-FU), an anticancer drug, to treat *P. aeruginosa* infections. Firstly, we investigated the inhibitory effects of 5-FU on bacterial growth using the microdilution method. Secondly, the impact of 5-FU on biofilm formation and disassembly was assessed via biofilm biomass measurements with the crystal violet staining method and confocal microscopy analyses. Lastly, the potential synergy between 5-FU and the antibiotics gentamicin and meropenem was evaluated using a checkerboard assay. Results revealed that 5-FU inhibited bacterial growth in a dose-dependent manner, with 100% inhibition observed at concentrations of 25 µg/mL and higher. Also, 70% and 100% reductions in biofilm biomass were demonstrated at concentrations of 12 and 100 µg/mL, respectively. Controversy, these higher concentrations unexpectedly increased biofilm biomass in pre-formed biofilms. Synergistic interactions were observed between 5-FU and gentamicin in both growth inhibition (FICI 0.31) and biofilm inhibition (ZIP 14.1), while no synergy was found with meropenem. These findings highlight the potential of 5-FU as an adjunctive therapy for *P. aeruginosa* infections, especially in combination with gentamicin. However, further research is required to address 5-FU limitations against mature biofilms.

## 1. Introduction

*Pseudomonas aeruginosa* is non-spore-forming, aerobic, and Gram-negative rod bacterium. It is found naturally in the environment and is associated with infections in both immunocompetent and immunocompromised hosts. It is also known to be involved in both acute and chronic wound infections, which are usually associated with increased morbidity and mortality rate. The highly adaptive strategies of this organism and its ability to form biofilm are key factors in enhancing its survival in hostile environments and increased resistance to antibiotic treatments [1,2]. By the year 2021, the Centers for Disease Control and Prevention listed multidrug-resistant *P. aeruginosa* as one of the top six threats in regard to antibiotic resistance. The World Health Organization lists it as one of the pathogens with an urgent need for the development of new antimicrobial agents [3,4].

Although antibiotic resistance in *P. aeruginosa* and other organisms is a public health crisis, the production of new antibiotics has been insufficient. Therefore, one advantageous area of research is the repurposing of approved drugs that were initially developed for purposes other than antimicrobial activity [5,6]. These agents have already undergone rigorous safety evaluations by health agencies and have well-characterized pharmacokinetic parameters, thus providing a cost- and time-effective method of drug discovery [7,8].

Nucleoside analogs are chemically modified purine and pyrimidine drugs and represent around 20% of the chemotherapeutics that are currently in use. Most nucleoside analogs mimic natural nucleic acid building blocks and can interfere with DNA/RNA synthesis in tumor cells. For over 60 years, 5-fluorouracil has been a fluoropyrimidine that has been used to treat tumors such as breast, colorectal, and pancreatic tumors. Like other fluoropyrimidines, this drug inhibits thymidylate synthase enzymes (TSases), which are essential for completing the last step of the de novo synthesis of thymidylate, which in turn impairs DNA replication and repair [9]. Interestingly, bacterial and mammalian TSases share notable similarities. Therefore, one attractive new way to battle antimicrobial organisms such as *P. aeruginosa* is repurposing 5-fluorouracil and using it to block DNA synthesis in replicating organisms. Despite this similarity, no antibacterial drug targeting TSases have been used [10].

It has been demonstrated that 5-fluorouracil can affect the growth and virulence factor production of both Gram-positive and Gram-negative organisms. One study found that 5-fluorouracil suppresses the production of pyoverdine, protease, and exotoxins in *P. aeruginosa* by inhibiting the expression of the iron-starvation gamma factor PvdS [11]. Additionally, this drug can inhibit the biofilm formation of *Escherichia coli* [12]. Moreover, several studies have reported that 5-fluorouracil can inhibit the growth of *P. aeruginosa* [13,14]. These findings demonstrate the possibility of using 5-fluorouracil to control *P. aeruginosa* infections.

The use of combination therapy is a promising strategy for enhancing treatment efficacy against infections caused by resistant bacteria. The effectiveness of drug combinations can be measured using methods like the checkerboard assay, which allows for the evaluation of drug interactions in vitro. With the growing prevalence of antibiotic-resistant strains of *P. aeruginosa,* exploring drug combinations through such methods can aid in the development of advanced therapeutic protocols [15]. In 2022, Nikolic et al. showed that combining phage JG024 with ciprofloxacin and administering it simultaneously or in succession with a short delay can have a synergistic effect against bacterial growth [16]. Domalaon et al. studied the possible synergy of a tobramycin–ciprofloxacin antibiotic hybrid and different anticancer agents. Using a checkerboard assay, they found synergistic effects with mitomycin C but not with 5-fluorouracil [17].

This study investigated the possibility of repurposing the anticancer drug 5-fluorouracil for treating *P. aeruginosa* infections in vitro. The focus was on testing the inhibitory effect of 5-fluorouracil on both the growth and formation of biofilms. Additionally, this research evaluated the possibility of using a checkerboard assay to investigate the potential synergy of 5-fluorouracil with meropenem and gentamicin.

## 2. Materials and Methods

### 2.1. Bacterial Strain, Drugs, and Growth Media

All experiments were performed using *P. aeruginosa* PAO1, which was kindly donated by Dr. Lee Hughes at the University of North Texas, Denton. The strain was stored at −80 °C in tryptic soy broth (SPML, Riyadh, Saudi Arabia) with 10% glycerol (Sigma-Aldrich, St. Louis, MO, USA). To activate the strain, it was subcultured on tryptic soy agar (SPML) and incubated aerobically at 37 °C for 18–20 h. Once an isolated colony was obtained, it was inoculated in 10 mL of *Pseudomonas* Minimal Media (PsMM), as described previously [18]. The organism suspension was used in experiments once it reached the mid-log phase with optical density (OD) readings ranging between 0.5 and 0.6 at 600 nm, as measured by a spectrophotometer (Libra S22, Biochrom Ltd., Cambridge, UK). Throughout the study, the concentration of 5-fluorouracil was considered inhibitory if it could achieve 80% inhibition or more according to the following: % inhibition = ((OD_untreated_ − OD_test_)/OD_untreated_) × 100 [19,20].

5-fluorouracil was obtained from Ebewe Pharma (FAREVA Unterach GmbH, Unterach am Attersee, Austria) as a powder, and a suspension was made according to the manufacturer’s guidelines to obtain a stock solution of 5 mg/mL. All 5-fluorouracil suspensions were made using sterile saline (Pharmaceutical solutions industry, Jeddah, Saudi Arabia). After suspension in saline, 5-fluorouracil can be stable at −5 °C for up to 28 days [21]. The antibiotics used for the checkerboard assay were gentamicin (Gibco by Life Technologies, Gran Island, New York, NY, USA) and meropenem (Ronem, Venus Remedies Ltd., Panchkula, India). Both drugs were obtained in powder form and reconstituted in distilled water to prepare stock solutions at a concentration of 50 mg/mL.

### 2.2. Antibacterial Activity

Antimicrobial activity was assessed using a microdilution broth susceptibility test according to the guidelines of the Clinical and Laboratory Standards Institute (CLSI) [22,23] with some modifications. In summary, several dilutions of 5-fluorouracil were prepared in a 96-well microtiter plate with increasing concentrations ranging from 0.1 to 500 μg/mL [24]. PsMM was used as the media, and 50 μL of bacterial suspension was added to each well, achieving a final volume of 200 μL per well. Next, the plate was incubated at 37 °C for 18–20 h.

To assess bacterial growth, the OD was measured at 600 nm using a BioTek Synergy HT microplate reader (BioTek Instruments, Winooski, VT, USA) [22]. Wells with and without bacterial cells were used as positive and negative controls, respectively. The initial OD at 0 h was subtracted from the post-incubation OD for all wells to account for baseline levels and accurately calculate the growth increase. For each concentration, the % inhibition was calculated. The OD values were measured relative to the untreated control to determine the reduction in growth in the test samples.

### 2.3. Synergy Testing Between 5-Fluorouracil and Gentamicin/Meropenem Using Checkerboard Assay

The synergistic efficacy of 5-fluorouracil in combination with antibiotics was tested by a checkerboard microdilution assay. The test was performed for two different combinations, 5-fluorouracil with gentamicin and 5-fluorouracil with meropenem. Briefly, the experiment was performed in 96-well plates with final volumes of 200 µL. Two-fold serial dilutions of 5-fluorouracil (0.125 to 32 µg/mL) were made across the *x*-axis, while the antibiotic was similarly diluted along the *y*-axis (0.125 to 8 µg/mL). Tests were conducted separately using gentamicin and meropenem [25]. This created a matrix where each well contained a combination of both 5-fluorouracil and each of the antibiotics with diverse concentrations. Next, 50 µL of bacterial cells grown in PsMM to the mid-log phase (OD_600_ = 0.5) were added.

The experiments were performed in triplicate with at least three controls included on each plate; 5-fluorouracil or antibiotic alone was used as the positive control and untreated cells were used as the negative control. The first row contained the serial dilutions of 5-fluorouracil alone, and the first column contained the serial dilutions of the antibiotic alone (either gentamicin or meropenem). The microplates were then incubated at 37 °C for 18–20 h, and then the absorbance of wells was read at 600 nm using the BioTek Synergy HT microplate reader.

The initial OD at 0 h was subtracted from the post-incubation OD for all wells to account for baseline levels and accurately calculate the growth increase. The fractional inhibitory concentration index (FIC) of 5-fluorouracil was calculated by dividing the MIC of the drug in the presence of the antibiotic by the MIC of the drug alone. Similarly, the FICs of gentamicin and meropenem were determined by dividing the MIC of the antibiotic in the presence of 5-fluorouracil by the MIC of the antibiotic alone. The MIC was defined as the lowest concentration of the drug/antibiotic that inhibited 80% of bacterial cell growth [26]. The FIC index (FICI) was then calculated as the sum of both FIC values. The FICI was interpreted as synergistic, additive, and antagonistic for values less than or equal to 0.5, between 0.5 and 4, and equal to or greater than 4, respectively [27].

### 2.4. Effect on Biofilm Biomass

The impact of 5-fluorouracil on the biofilm biomass of PAO1 was evaluated using 6-well Greiner plates. A uniform methodology was applied for biofilm biomass, biofilm eradication, and live/dead assays to ensure consistency across experiments. As previously described by Gnanadhas et al. [28], biofilms of PAO1 were grown in 6-well plates by adding bacterial culture to PsMM media up to a total volume of 1 mL. The concentrations of 5-fluorouracil tested were 100, 12, 0.5, and 0.1 µg/mL. The plates were incubated in static conditions at 37 °C for 48 h. After incubation, the wells were washed twice with distilled water to remove planktonic cells. During this washing step, precautions were taken to avoid damage to the biofilm.

Next, 1.2 mL of 0.5% crystal violet (CV) solution (Sigma-Aldrich) were added to each well. After 20 min of incubation at room temperature, the excess CV was washed away, and 95% ethanol (Sigma-Aldrich) was added to dissolve the remaining dye. Absorbance was measured at 490 nm using a BioTek Synergy HT microplate reader. The % inhibition was calculated for each concentration. The inhibitory concentration was determined to be the concentration at which there was significant reduction in biofilm biomass measured using the OD at 490 nm compared to the untreated well.

### 2.5. Biofilm Eradication Assay

Biofilms of PAO1 were cultivated in 6-well plates by adding bacterial culture to PsMM media up to a final volume of 1 mL. The plates were maintained under static conditions for 48 h at 37 °C. Upon completion of incubation, the contents of each well were carefully removed and replaced by fresh PsMM media and 5-fluorouracil at the same concentrations used for the biomass studies (100, 12, 0.5, 0.1 µg/mL).

The plates were further incubated at 37 °C for an additional 24 h. Following this, the wells were rinsed twice with distilled water to remove any remaining planktonic cells. Disruption of the biofilm was carefully avoided during the washing step. Then, 1.2 mL of 0.5% CV solution (Sigma-Aldrich) was added to each well. After 20 min of incubation at room temperature, excess CV was removed, and 95% ethanol (Sigma-Aldrich) was used to remove the residual stain. The absorbance of samples was determined at 490 nm using the BioTek Synergy HT microplate reader [29].

### 2.6. Effects on Biofilm Morphology

The effects of 5-fluorouracil on biofilms of PAO1 were assessed with confocal laser scanning microscopy (CLSM) (Nikon C2, Nikon Instruments Inc., Tokyo, Japan). This assay was conducted as described previously [30]. PAO1 was cultured on coverslips in 6-well culture plates containing media with 0.1, 0.5, 12, and 100 µg/mL of 5-fluorouracil concentrations. The plate was incubated at 37 °C for 48 h to allow biofilms to form on the coverslips.

After 48 h, the broth was gently removed from each well, and any remaining planktonic cells were removed by washing three times with phosphate-buffered saline (PBS). The coverslips were then carefully transferred to a new 6-well plate, and approximately 200 μL of LIVE/DEAD™ BacLight™ working solution (Invitrogen Ltd., Paisley, UK) was added onto each coverslip according to the manufacturer’s instructions, followed by incubation for 30 min at room temperature in the dark. The morphology of the biofilms that formed with and without 5-fluorouracil was studied using CLSM. The excitation/emission was 488 nm/<550 nm for SYTO 9 (BacLight™ Component A) and 568 nm/>600 nm for propidium iodide (BacLight™ Component B).

Images were captured and three-dimensional plots of the biofilm samples were constructed with NIS-Elements Advanced Research Software (version 4.0, Nikon, Tokyo, Japan) [31]. Image analysis was carried out using ImageJ software (version 1.50i, NIH, Bethesda, Maryland) [31]. The percentage of dead PAO1 cells was determined by calculating the ratio of red fluorescence (indicating dead bacteria) to the total fluorescence (green for live bacteria and red for dead bacteria) in each image. This value was then multiplied by 100 to obtain the death percentage according to a reported method [32]. The inhibitory effect of different concentrations of 5-fluorouracil after treatment on the *P. aeruginosa* biofilm depth and formation were visualized and evaluated on the generated sample optical slides [31].

### 2.7. Synergy Testing of Drug Combination Against P. aeruginosa Biofilms

The possible synergy of the drug combinations on *P. aeruginosa* biofilm inhibition was assessed using the same pairings and methodology as the checkerboard assay. After inoculation, the biofilms were incubated at 37 °C for 48 h in static conditions. Post-incubation, biofilm biomass was quantified using the crystal violet (CV) staining method, as previously described in the biofilm protocol. For data analysis, the dose–response matrix was analyzed using the Synergy Finder online tool (version 3.0), and the synergistic interaction was quantified based on the zero interaction potency (ZIP) model [32]. The study focused on two drug combinations, gentamicin with 5-fluorouracil and meropenem with 5-fluorouracil.

### 2.8. Statistical Analysis

For each study, a minimum of three independent experiments were performed. Significant differences between untreated and treated samples were determined by one-way analysis of variance (ANOVA) using GraphPad Prism software (version 10.3.1 (464)), followed by a pairwise comparison procedure using Dunnett’s multiple comparisons test. In all cases, statistical significance was defined by a *p* value of <0.05.

## 3. Results

### 3.1. Antibacterial Effects

**Figure 1:**
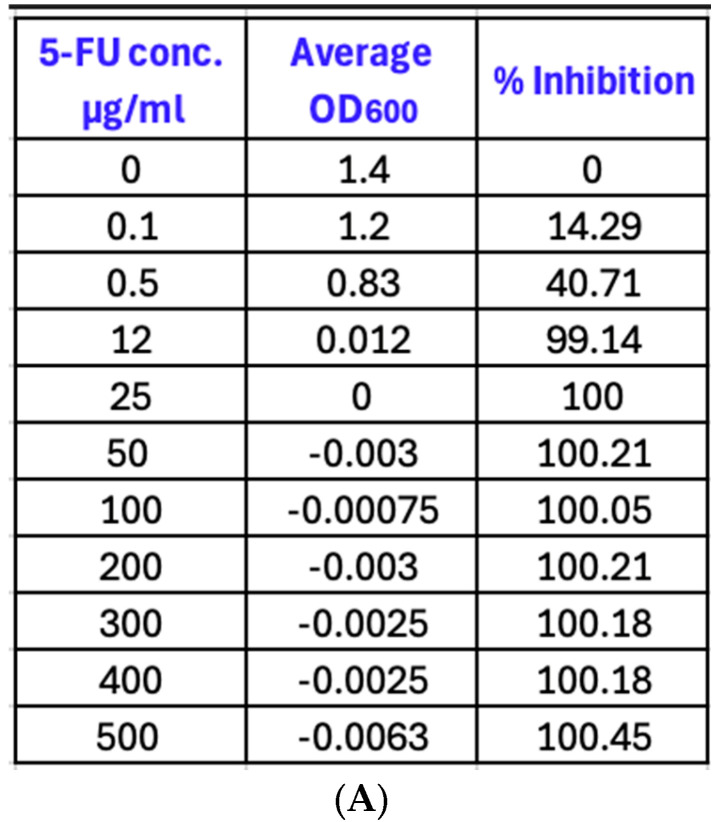

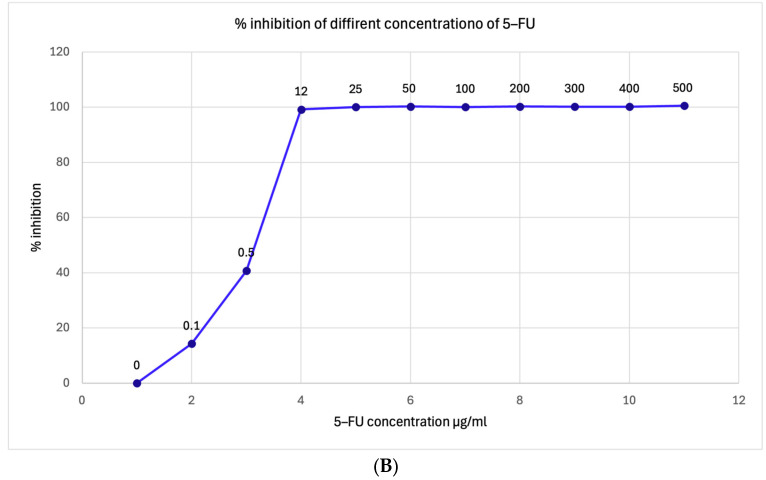
Efficacy of different concentrations of 5-fluorouracil on the growth of PAO1. (**A**) Average OD_600_ reading and % inhibition of 5-fluorouracil concentrations ranging from 0 to 500 µg/mL; (**B**) % inhibition of bacterial growth at different concentrations of 5-fluorouracil. The potency of the drug decreased significantly at concentrations below 0.5 µg/mL, while it remained above 95% at concentrations of 12 µg/mL and above. Data points represent the mean values of at least 3 replicates. For all concentrations, there was a significant decrease in growth with a *p* value of 0.0044 (*p* < 0.05) for a concentration of 0.1 µg/mL and *p* < 0.0001 for concentrations of 0.5 to 500 µg/mL.

The percent inhibition was calculated for each concentration to determine the MIC. The results showed that 5-fluorouracil had high inhibition levels of 100% at concentrations of 25, 50, 100, 200, 300, 400, and 500 µg/mL. At a concentration of 0.5 µg/mL, the ability of 5-fluorouracil to inhibit the growth of PAO1 dropped to almost 41% and declined significantly from this point, reaching a low point of 14.25% inhibition at 0.1 µg/mL (Figure 1).

### 3.2. Checkerboard Assays

**Figure 2:**
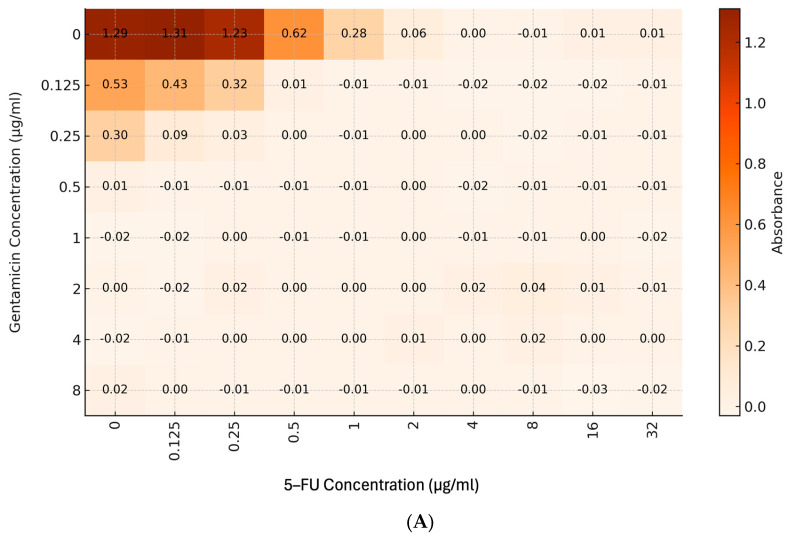

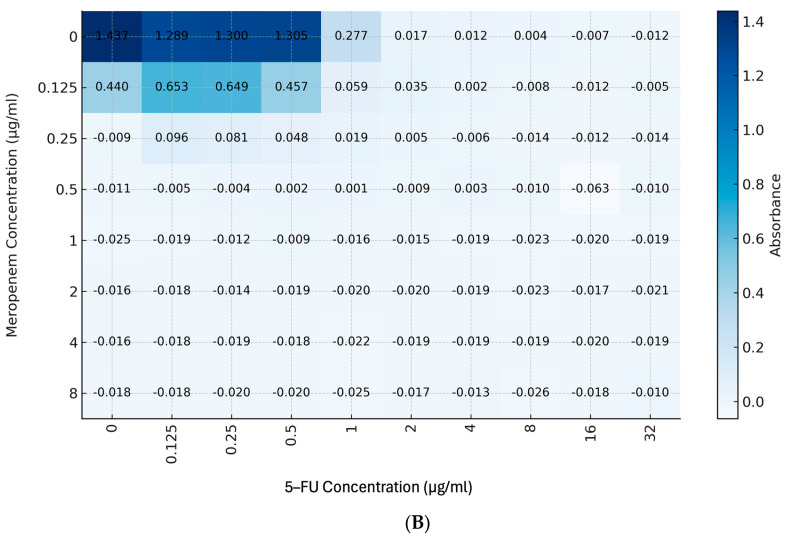
Heatmap displaying the OD_600_ readings from a checkerboard assay in a 96-well plate (horizontal axis, µg/mL) and gentamicin (vertical axis, 0–8 µg/mL). A darker color represents a higher increase in absorbance, which corresponds to increased bacterial growth. The FICI was calculated from each drug with the MIC (the lowest concentration of the drug showing 80% inhibition). (**A**) Combined treatment of gentamicin and 5-fluorouracil indicated synergy with an IFCI value of 0.31. (**B**) Combined treatment of meropenem and 5-fluorouracil indicated an additive effect with an FICI value of 0.56.

5-fluorouracil was tested in combination with gentamicin for potential synergy against PAO1. Gentamicin exhibited synergy with 5-fluorouracil with an FICI of 0.31. Meropenem showed an additive effect with an FICI of 0.56 (Figure 2).

### 3.3. Biofilm Biomass

**Figure 3:**
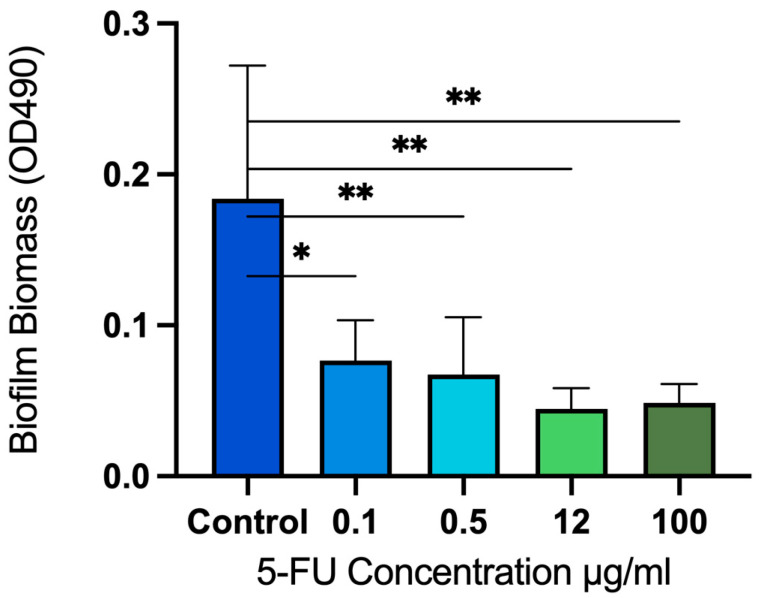
Absorbance measurements at 490 nm showing biofilm biomass at different concentrations of 5-fluorouracil. Values are adjusted for background absorbance. 5-fluorouracil significantly reduced biofilm biomass compared to the untreated control group with a maximum reduction observed at concentrations of 12 and 100 µg/mL. * *p* < 0.01; ** *p* < 0.001.

Compared to the untreated sample, a significant decrease in the biofilm biomass was observed at all concentrations of 5-fluorouracil with *p* < 0.05 at a concentration of 0.1 µg/mL and *p* < 0.005 for concentrations of 0.5, 12, and 100 µg/mL. The percentage of inhibition of biofilm biomass was calculated as 58% and 63% at concentrations of 0.1 and 0.5 µg/mL, respectively. At concentrations of 12 and 100 µg/mL, 5-fluorouracil inhibited biofilm formation by over 70% (Figure 3).

### 3.4. Disassembly of Established Biofilm

**Figure 4:**
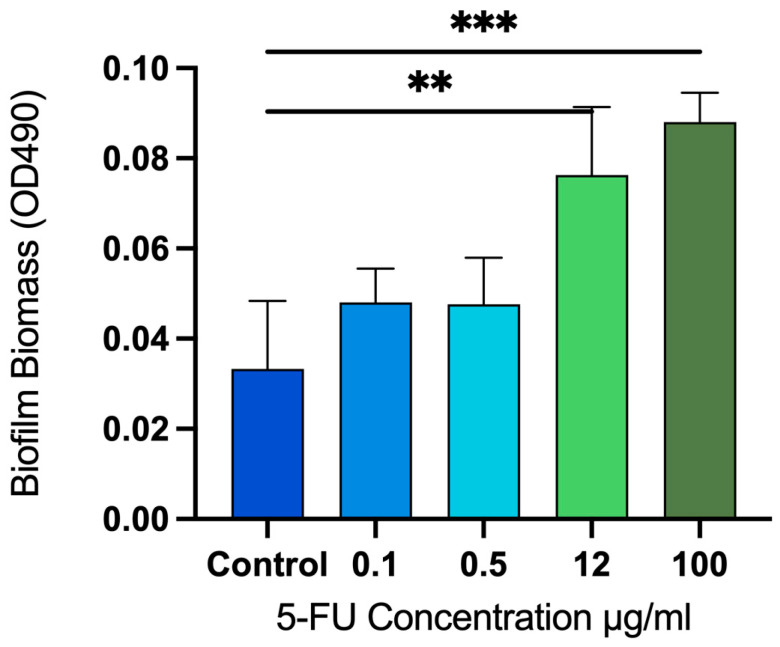
Absorbance measurements at 490 nm showing biofilm dispersion after the addition of different concentrations of 5-fluorouracil. Values are adjusted for background absorbance. Established biofilms were treated with increasing concentrations of 5-fluorouracil (0.1, 0.5, 12, and 100 µg/mL) for 24 h. 5-fluorouracil resulted in a dose-dependent increase in biofilm biomass rather than disassembly. ** *p* < 0.01; *** *p* < 0.001.

The 5-fluorouracil did not appear to have the ability to disassemble an established biofilm. Instead, it increased the biofilm biomass when used at higher concentrations. There was no significant change in biofilm biomass at concentrations of 0.1 and 0.5 µg/mL of 5-fluorouracil when compared to the untreated control sample. However, at concentrations of 12 and 100 µg/mL, it caused a significant increase in biofilm biomass, *p* < 0.05. The percentage increase in biofilm biomass at the concentrations of 12 and 100 µg/mL was calculated relative to the untreated control group as 129.10% and 164.86%, respectively (Figure 4).

### 3.5. Biofilm Formation

**Figure 5:**
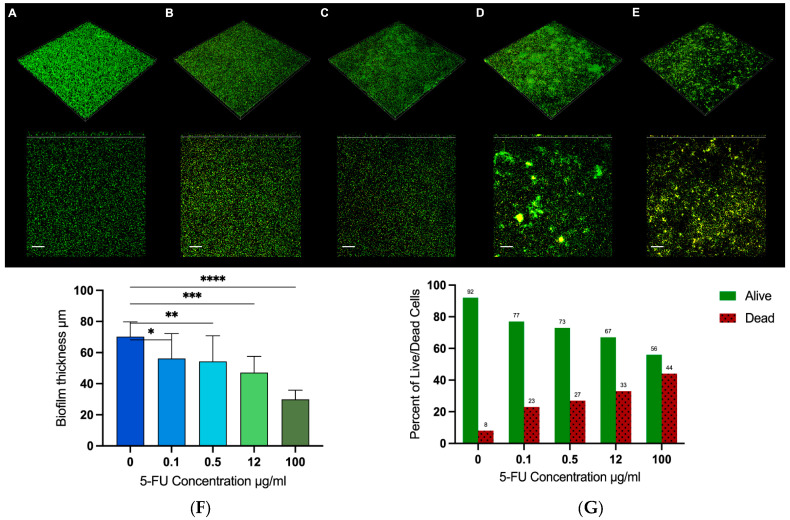
Dose-dependent effect of 5-fluorouracil on *P. aeruginosa* biofilms. Representative CLSM images of PAO1 biofilm 48 h after treatment with 5-fluorouracil (100 µm scale). Biofilms obtained with (**A**) untreated control and treatments with (**B**) 0.1, (**C**) 0.5, (**D**) 12, and (**E**) 100 µg/mL 5-fluorouracil. (**F**) Biofilm thickness measurement expressed in µm. (**G**) Cell viability graph showing the percentage of live cells (green bars) and dead cells (red bars). Data are expressed as the mean. * *p* < 0.05; ** *p* < 0.01; *** *p* < 0.001; ****p < 0.0001.

A significant dose-dependent increase in dead cells and a corresponding decrease in live cells were observed across all tested concentrations (*p* < 0.0001). The percentage of dead cells in the untreated group was 8%, while it increased to 23%, 27%, 33%, and 44% at concentrations of 0.1, 0.5, 12, and 100 µg/mL, respectively. Concurrently, there was a corresponding dose-dependent decrease in the percentage of live cells. That of the control group was 92%, and the values dropped to 77%, 73%, 67%, and 56% at concentrations of 0.1, 0.5, 12, and 100 µg/mL, respectively. The biofilm thickness measured by CLSM showed a decreasing trend with increasing concentrations of 5-fluorouracil. The measurements were 70 µm at 0 µg/mL, 56 µm at 0.1 µg/mL, 55 µm at 0.5 µg/mL, 47 µm at 12 µg/mL, and 31 µm at 100 µg/mL (Figure 5).

### 3.6. Efficacy of the Antibiotic and 5-Fluorouracil Combination Against P. aeruginosa Biofilm

**Figure 6:**
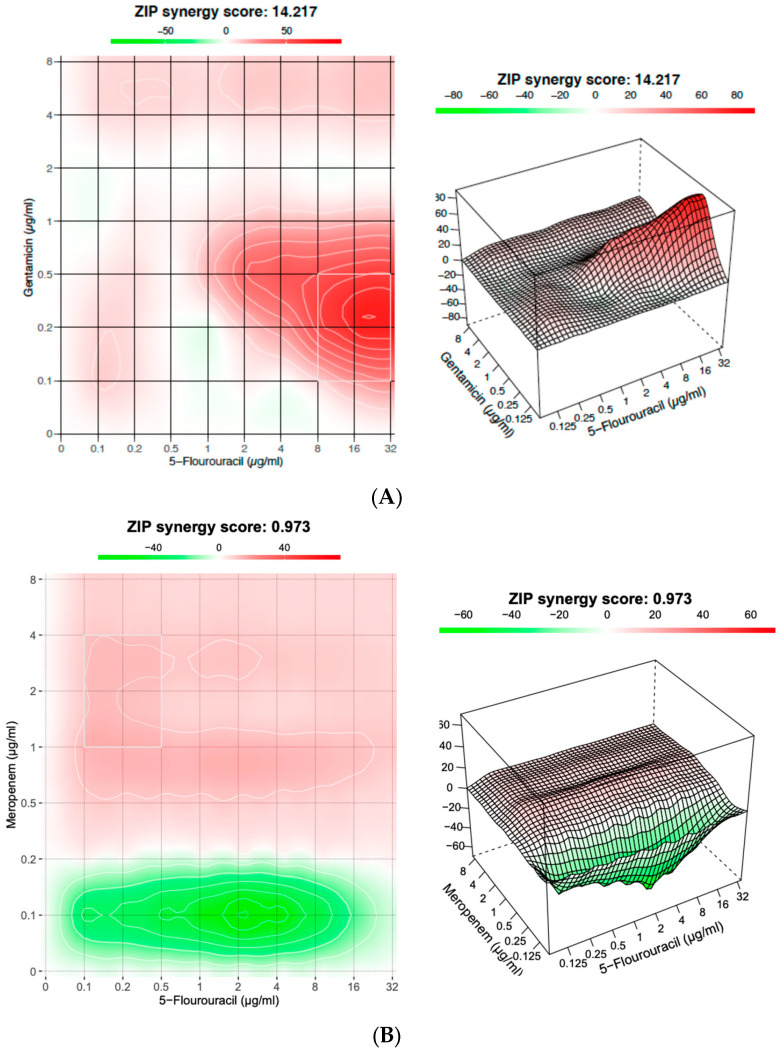
The average synergy score for a drug combination is determined based on all the dose combination measurements. The 2D and 3D synergy maps use red and green colors to indicate synergistic and antagonistic dose regions, respectively. (**A**) Gentamicin and 5-fluorouracil ZIP synergy score of 14.2 (>10), likely indicating synergy. (**B**) Meropenem and 5-fluorouracil ZIP synergy score of 0.97 (<10), indicating no synergy.

The combination of gentamicin and 5-fluorouracil exhibited a ZIP synergy score of approximately 14.2, likely indicating synergy between the two drugs. In contrast, the combination of meropenem and 5-fluorouracil did not demonstrate synergy, with a ZIP score of 0.97. (Figure 6).

## 4. Discussion

The strategy of repurposing existing drugs that were originally developed and approved for other diseases is becoming increasingly attractive. This approach leverages the known safety profile and established mechanisms of action of these drugs and opens a valuable new avenue for the rapid development of specific antibacterial agents [33]. 5-fluorouracil has been described as a growth inhibitor for Gram-negative organisms [13] and a growth and biofilm inhibitor for Gram-positive organisms [34]. The present study investigated its efficacy with a focus on its effects on antibacterial activity, biofilm inhibition, biofilm disassembly, and viability assays to treat *P. aeruginosa* infections.

**Figure 7:**
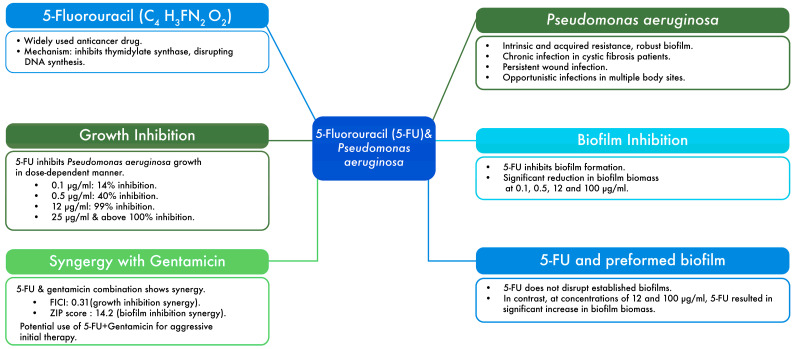
Overview of the key findings of this study.

The findings of this study are summarized in Figure 7, which highlights the synergy between 5-fluorouracil and gentamicin, as well as the limitations of 5-fluorouracil in disrupting pre-formed biofilms. The results revealed that inhibition of *P. aeruginosa* growth by 5-fluorouracil is dose-dependent. This could be attributed to the compound’s hydrophilic nature, which allows efficient passage through outer bacterial membranes via porins, which increases its concentration in the bacterial cytoplasm, where it can interfere with DNA and RNA synthesis. Therefore, higher concentrations of 5-fluorouracil lead to a greater inhibition of bacterial growth [35].

Using the broth microdilution method, we found that 5-fluorouracil can produce almost a 100% inhibition of bacterial growth at varying concentrations ranging from 12 to 100 µg/mL. After that point, the % inhibition decreases to about 50% and 16% at concentrations of 0.5 and 0.1 µg/mL, respectively. These concentrations are lower than those reported by Domalaon et al., who indicated an MIC of 64 µg/mL [17]. The different susceptibilities of *P. aeruginosa* strains could be attributed to the differing experimental conditions—specifically, the use of a growth medium tailored to *P. aeruginosa* in our study—which might explain this variation, as it could affect the uptake of 5-fluorouracil by the bacteria [36].

It is hypothesized that the antibacterial activity of 5-fluorouracil could be further augmented by combination with clinically used antibacterials that target different areas of the bacterial cell. For example, gentamicin interferes with protein synthesis, and meropenem inhibits cell wall synthesis. The use of another substance to augment antibiotics has been proven effective both in vitro and in vivo. For instance, Engeman et al. reported that the use of a phage–antibiotic combination resulted in increased susceptibility in multidrug-resistant *P. aeruginosa* strains and reduced the burden of the infection in mouse wound models [37,38]. Other studies demonstrated that combinations of silver nanoparticles and antibiotics have significantly higher antibacterial activity against bacteria [39,40]. In the current study, 5-fluorouracil showed no synergy with meropenem. On the other hand, it demonstrated a synergistic effect when combined with gentamicin in vitro. And gentamicin showed promising results, demonstrating synergy with 5-fluorouracil in both inhibiting bacterial growth and reducing biofilm formation. In contrast, meropenem did not exhibit synergy for biofilm inhibition and had an additive effect in inhibiting bacterial growth. The observed synergy between 5-fluorouracil and gentamicin may be contributed to by their distinct mechanisms of action targeting separate essential processes in the bacterial cell. 5-fluorouracil interferes with thymidylate synthase, disrupting DNA synthesis within bacterial cells. This interference impairs bacterial replication and reduces cellular viability. Meanwhile, gentamicin binds to the 30S ribosomal subunit, leading to mRNA misreading and defective protein synthesis. This dual-action technique complicates the ability of the bacteria to simultaneously build resistance to both medications, enhancing the combination’s overall therapeutic efficacy. Further research is necessary to investigate how this relationship might be utilized to combat *P. aeruginosa* infections.

The ability of *P. aeruginosa* to form intrinsically resistant biofilms presents another major threat to human health, as several infections are directly related to biofilm formation, such as lung infections in individuals with cystic fibrosis and persistent wound infections. Therefore, this study also focused on understanding the effect of 5-fluorouracil on the biofilm formation of *P. aeruginosa*. The results showed that 5-fluorouracil effectively reduces biofilm formation by reducing both the thickness and biomass. The reduction in biofilm biomass was dose-dependent, ranging from a 58% reduction at a 5-fluorouracil concentration of 0.1 µg/mL to 70% at 100 µg/mL. These findings agree with results from other studies, which reported a reduction in the biofilm of *P. aeruginosa* at a 5-fluorouracil concentration of 60 µg/mL [41,42]. This reduction in biomass could be attributed to the killing of bacterial cells, as the CLSM live–dead assay revealed that the percentage of dead cells increased from 8% in the untreated group to 44% in the group treated with 100 µg/mL of 5-fluorouracil. These observations are consistent with the results obtained from the CV method. Interestingly, confocal microscopy imaging revealed increased aggregation at high concentrations (12 and 100 µg/mL), which likely represents a stress response by the bacteria to survive the drug’s effects. This aggregation was evident in the imaging results, even as we noted a decrease in the overall biofilm biomass and thickness. This is worth investigating further, as aggregates are known to be as robust as biofilms in their ability to enable bacterial infections to persist and resist treatment [43,44].

When 5-fluorouracil was tested on a 48 h pre-formed biofilm, it was unable to disperse the biofilm. Instead, it resulted in an increase in biofilm biomass at the same concentrations of 12 and 100 µg/mL. These findings underscore the complex interaction between the drug and the bacteria, warranting further investigation. This suggests a potential defensive response by the bacteria to counteract the toxic effects of 5-fluorouracil. This can be investigated further by examining the expression of genes involved in biofilm formation during the initial establishment versus after the biofilm is fully formed. Another interesting area of research involves structural modifications to 5-fluorouracil. Specifically, studies have shown that the addition of phosphonium groups improves the drug’s antimicrobial and antibiofilm activity. These modifications suggest that 5-fluorouracil’s effectiveness can be further enhanced through targeted structural changes, potentially enabling it to disrupt mature biofilms [14]. While we acknowledge this limitation, this result does not diminish the potential of 5-fluorouracil in combating *P. aeruginosa* infections. Instead, it highlights the need for further research on its possible use, particularly as part of an early-stage treatment strategy—possibly in combination with gentamicin—to enhance bacterial clearance and prevent the development of chronic infections.

This study has some limitations. First, the findings are based on in vitro experiments, and no testing on clinical isolates or resistant *P. aeruginosa* strains was performed. Second, more detailed investigations are required to explore the cellular interactions between 5-fluorouracil and the bacteria to understand and address the inability of 5-fluorouracil to disrupt mature biofilms, which in turn highlights the challenge of using this drug in the later stages of infection. Third, the synergistic effect of 5-fluorouracil and gentamicin should be evaluated using animal models, such as wound infection models, to assess its therapeutic potential.

## 5. Conclusions

5-fluorouracil demonstrates significant potential as an initial treatment for *P. aeruginosa* infections by reducing viable cells and inhibiting early biofilm formation. Additionally, it demonstrated a synergistic effect when used in combination with gentamicin. Nevertheless, its effectiveness diminished against mature 48 h biofilms and may even promote biofilm growth at higher concentrations. These findings are significant and call for further testing on how 5-fluorouracil affects the growth and biofilm formation of *P. aeruginosa*, which could pave the way for its use as a potential treatment.

## Data Availability

The original contributions presented in the study are included in the article, further inquiries can be directed to the corresponding author.
